# Development and Evaluation of a Mental Health Chatbot Using ChatGPT 4.0: Mixed Methods User Experience Study With Korean Users

**DOI:** 10.2196/63538

**Published:** 2025-01-03

**Authors:** Boyoung Kang, Munpyo Hong

**Affiliations:** 1 Sungkyunkwan University Seoul Republic of Korea

**Keywords:** mental health chatbot, Dr. CareSam, HoMemeTown, ChatGPT 4.0, large language model, LLM, cross-lingual, pilot testing, cultural sensitivity, localization, Korean students

## Abstract

**Background:**

Mental health chatbots have emerged as a promising tool for providing accessible and convenient support to individuals in need. Building on our previous research on digital interventions for loneliness and depression among Korean college students, this study addresses the limitations identified and explores more advanced artificial intelligence–driven solutions.

**Objective:**

This study aimed to develop and evaluate the performance of HoMemeTown Dr. CareSam, an advanced cross-lingual chatbot using ChatGPT 4.0 (OpenAI) to provide seamless support in both English and Korean contexts. The chatbot was designed to address the need for more personalized and culturally sensitive mental health support identified in our previous work while providing an accessible and user-friendly interface for Korean young adults.

**Methods:**

We conducted a mixed methods pilot study with 20 Korean young adults aged 18 to 27 (mean 23.3, SD 1.96) years. The HoMemeTown Dr CareSam chatbot was developed using the GPT application programming interface, incorporating features such as a gratitude journal and risk detection. User satisfaction and chatbot performance were evaluated using quantitative surveys and qualitative feedback, with triangulation used to ensure the validity and robustness of findings through cross-verification of data sources. Comparative analyses were conducted with other large language models chatbots and existing digital therapy tools (Woebot [Woebot Health Inc] and Happify [Twill Inc]).

**Results:**

Users generally expressed positive views towards the chatbot, with positivity and support receiving the highest score on a 10-point scale (mean 9.0, SD 1.2), followed by empathy (mean 8.7, SD 1.6) and active listening (mean 8.0, SD 1.8). However, areas for improvement were noted in professionalism (mean 7.0, SD 2.0), complexity of content (mean 7.4, SD 2.0), and personalization (mean 7.4, SD 2.4). The chatbot demonstrated statistically significant performance differences compared with other large language models chatbots (*F*=3.27; *P*=.047), with more pronounced differences compared with Woebot and Happify (*F*=12.94; *P*<.001). Qualitative feedback highlighted the chatbot’s strengths in providing empathetic responses and a user-friendly interface, while areas for improvement included response speed and the naturalness of Korean language responses.

**Conclusions:**

The HoMemeTown Dr CareSam chatbot shows potential as a cross-lingual mental health support tool, achieving high user satisfaction and demonstrating comparative advantages over existing digital interventions. However, the study’s limited sample size and short-term nature necessitate further research. Future studies should include larger-scale clinical trials, enhanced risk detection features, and integration with existing health care systems to fully realize its potential in supporting mental well-being across different linguistic and cultural contexts.

## Introduction

The COVID-19 pandemic has exacerbated the already concerning rates of depression and anxiety among college students worldwide [1,2]. In Korea, the situation is particularly alarming. Recent statistics highlight the severity of mental health issues among college students in this country. According to the “2021 COVID-19 National Mental Health Survey,” individuals in their 20s showed the highest average depression score Patient Health Questionnaire-9 (PHQ-9) of 6.7 and the highest proportion of high-risk depression groups at 30% among all age groups surveyed. This represents a significant increase from 2018 when the average depression score was 2.3. The proportion of the high-risk depression group (PHQ-9 score ≥10) increased to 22.8% in 2021, approximately 6 times higher than in 2018.

Our previous study [3] on digital interventions for loneliness and depression among Korean college students highlighted the need for more personalized and culturally sensitive approaches, which this research aims to address. Among the initial 63 applicants in that study, the average PHQ-9 score was 9.23, with 25 (39.7%, 25/63) participants classified as high-risk for depression (PHQ-9 score ≥10). At the baseline of our study with 53 participants, 23 (43.4%, 23/53) were categorized as high-risk for depression. These findings underscore the urgent need for effective interventions to improve mental health among college students in Korea.

Despite the increasing severity of mental health issues among college students, the infrastructure and support systems within universities to effectively address these problems remain inadequate. In Korea, the annual mental health budget for college students is limited, with most of the resources focused on counseling services and little emphasis on preventive approaches (Counseling Council for University Students, Furthermore, due to the lack of professional counseling personnel and resources, access to services is limited, and many students are unable to receive timely and appropriate help [4,5].

Social stigma and prejudice against mental health issues also act as significant barriers for college students seeking help. Many students, despite experiencing psychological difficulties, tend to avoid help-seeking behaviors due to negative perceptions about psychiatric treatment or counseling [6]. This can exacerbate symptoms and prolong problems. In particular, those with low mental health literacy are less likely to recognize their condition or understand the need for professional intervention. In fact, the mental health literacy score of college students who participated in our previous study [3] averaged only 2.57 out of 5 points, highlighting the urgent need for educational intervention in this area.

In this context, digital technology-based mental health management solutions are gaining attention as a new alternative. Digital intervention services that overcome spatial and temporal constraints and ensure anonymity can contribute to improving accessibility and participation. Considering the high digital literacy of college students, these methods can be more familiar and acceptable to them [7]. Recent advancements in artificial intelligence (AI) and natural language processing (NLP) have paved the way for the development of sophisticated chatbots that can engage in human-like conversations and provide personalized support [2]. Large language models (LLMs), such as ChatGPT, have revolutionized the field of conversational AI. These models are trained on vast amounts of text data, enabling them to generate human-like responses and understand context. In mental health support, LLMs can be fine-tuned to provide empathetic responses, recognize emotional cues, and offer personalized support, making them potentially powerful tools for accessible mental health interventions.

Building upon the findings of our previous study [3], which explored the effectiveness of digital interventions for loneliness and depression among college students, this research aims to address the limitations identified in earlier digital interventions and develop a more effective and user-friendly mental health support tool. Specifically, this study focuses on the development and evaluation of an LLM-based chatbot prototype, named HoMemeTown, designed to provide personalized mental health support. The HoMemeTown chatbot, powered by ChatGPT 4.0, offers several unique features, that are (1) cross-lingual capability in English and Korean, ensuring cultural sensitivity, (2) a built-in gratitude journaling feature to promote positive thinking, (3) emotion recognition and empathetic response generation, and (4) risk detection algorithms to identify potential mental health crises.

These features aim to provide a comprehensive, user-friendly mental health support tool for young adults.

This pilot study was conducted as part of a larger research project titled “Development of a Youth Mental Health Platform Using Natural Language Processing.” While the overarching project received initial institutional review board approval, we acknowledge that this specific chatbot experiment was added later due to rapid developments in AI technology. Despite this limitation, we maintained rigorous ethical standards throughout our research, including informed consent, data privacy measures, and risk mitigation strategies.

The primary objective of this study is to conduct an initial usability test of the chatbot prototype, providing valuable insights for future, more comprehensive clinical studies. While our sample size is limited, we have used a mixed methods approach, combining quantitative usability metrics with in-depth qualitative feedback. This approach allows us to gain rich insights into user experiences and chatbot performance, even with a smaller participant pool. In addition, we have conducted comparative analyses with existing digital mental health tools to contextualize our findings within the broader landscape of mental health technologies.

As we navigate the rapidly evolving landscape of AI and its potential to revolutionize mental health support, it is crucial to explore innovative solutions that can bridge the gap between technology and human empathy [8]. This pilot study contributes to the growing body of knowledge surrounding the use of AI in mental health and sheds light on the potential of LLM-based chatbots like HoMemeTown to make a positive impact on people’s lives, while also identifying areas for future research and development.

## Methods

### Study Design and Participants

This pilot study used a mixed methods approach to evaluate the HoMemeTown chatbot’s usability and effectiveness in providing mental health support. In total, 20 participants (12 female and 8 male) aged 18-27 (mean 23.3, SD 1.96) years were recruited through university email lists and social media advertisements. The sample size was determined to be appropriate for this pilot study, particularly given that 70% (14/20) of participants had previous experience with mental health chatbots from our previous research [3], providing valuable comparative insights. This continuity in participation enhanced our ability to gather meaningful longitudinal observations about user engagement with mental health technologies.

Participant eligibility criteria were established to ensure appropriate sampling while maintaining ethical considerations. Eligible participants included university students aged 18-27 years with Korean language proficiency and access to digital devices. We excluded individuals with severe mental health conditions requiring immediate professional intervention, as determined through initial screening questionnaires. This exclusion criterion was implemented to ensure participant safety and appropriate levels of support, following established ethical guidelines in digital mental health research [9].

### Prototype Development Using the ChatGPT Application Programming Interface

The HoMemeTown chatbot is an innovative web-dependent service designed to support users in cultivating gratitude practice and providing emotional support through engaging, personalized interactions [10]. By leveraging cutting-edge NLP and emotion detection technologies, the HoMemeTown chatbot creates a unique and rewarding user experience that encourages regular engagement and promotes mental well-being [11].

The development process involved several key steps, including server setup, domain acquisition, Secure Sockets Layer certification, screen planning, development method selection, application programming interface (API) integration, front-end and back-end development, database design, performance tuning, and additional feature implementation [12].

The chatbot relies on the GPT API, a general-purpose language model provided by OpenAI, instead of a domain-specific model trained for mental health counseling. The GPT API offers a range of models, such as Davinci, GPT-3.5, and GPT-4, which can be selected based on desired performance and cost considerations. The chatbot relies on the GPT API, a general-purpose language model provided by OpenAI, instead of a domain-specific model trained for mental health counseling. The GPT API offers a range of models, such as Davinci, GPT-3.5, and GPT-4, which can be selected based on desired performance and cost considerations.

Rate limiting and resource management are (1) maximum 4,000,000 tokens per minute processing capacity, (2) up to 5000 requests per minute, (3) implementation of a request queuing system to prevent rate limit exceedance, and (4) monthly budget monitoring and automated alerts for cost control.

These technical constraints were carefully managed to ensure consistent service delivery while maintaining cost-effectiveness. Regular monitoring of API performance and reliability metrics helped optimize the system’s operation throughout the study period.

The desired functionalities of the chatbot, such as its role as a counselor and its ability to detect risks, are implemented through the use of prompts [8]. However, due to the nature of the GPT model, there is no guarantee that the chatbot will always behave exactly as intended, as its responses may vary slightly even with the same prompt [7].

**Figure 1:**
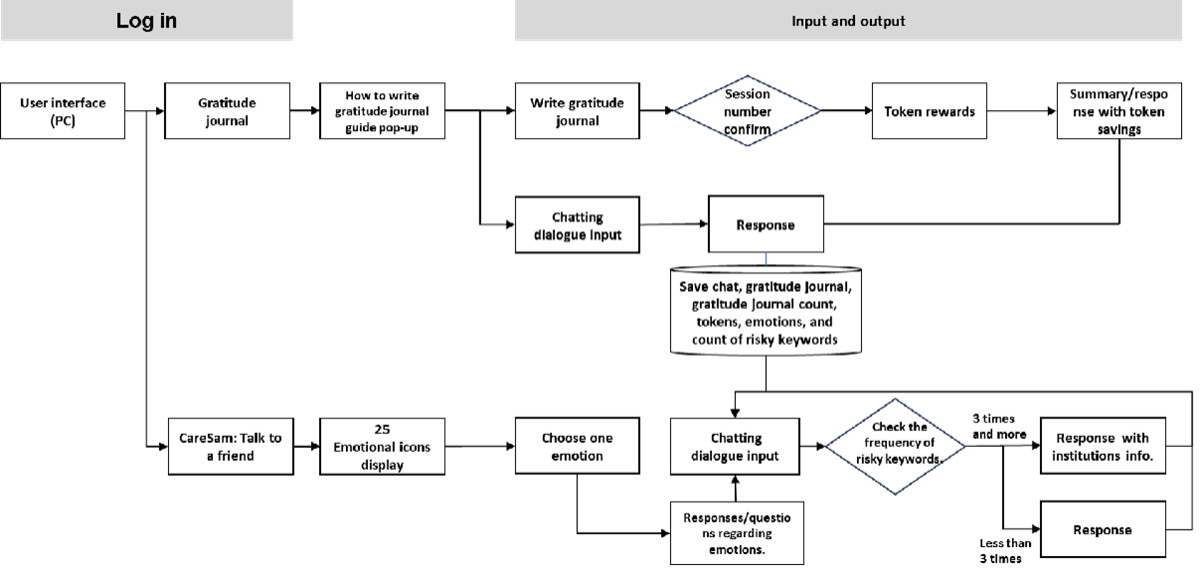
Service Flow Architecture of the HoMemeTown chatbot.

Figure 1 illustrates the service flow architecture of the HoMemeTown chatbot. The architecture depicts the user’s journey, starting from the login process through the user interface on their PC. After logging in, users can access the gratitude journal section, where they can find a guide on “How to write gratitude journal” and proceed to write their own entries [13]. The system assigns a unique session number to each journaling session and securely saves the user’s journal entries along with metadata such as the gratitude journal count, detected tokens or keywords, expressed emotions, and word count [14].

Users are rewarded with token rewards upon completing a journal entry, and the system generates a personalized response acknowledging their entry [4]. They can then continue their gratitude practice by initiating a new chat through the “CareSam: Talk to a friend” option. This feature allows users to select an emotion from a set of 25 emotional icons and provide more context about their feelings. Cowen and Keltner’s [15] research on emotional classification inspired the inclusion of these icons. Recognizing these 25 unique emotions can help users cultivate greater self-awareness and sensitivity toward others, leading to increased empathy, connection, and understanding.

The HoMemeTown chatbot aims to encourage and motivate users to cultivate gratitude practice by providing a seamless user flow, personalized responses, and emotional attunement.

The HoMemeTown chatbot is currently accessible as an open web application. This pilot version is available for public testing, allowing anyone to interact with the chatbot and experience its features firsthand. The chatbot’s continued operation demonstrates our commitment to transparency and ongoing exploration of digital mental health interventions. This open access approach enhances research reproducibility and provides opportunities for continuous feedback and improvement.

### Technical Implementation and Server Architecture

The HoMemeTown chatbot operates on a cloud infrastructure that prioritizes privacy through a “privacy by design” approach. The key technical feature is that no personal data or chat history is stored, making the system completely stateless between sessions (Textbox 1).

This minimalist architecture was specifically chosen to eliminate privacy concerns by avoiding any form of user data collection or storage. The system operates on a request-response basis, where each interaction is treated as a new session without any historical context or user identification. This approach, while limiting some personalization features, ensures maximum privacy protection for users engaging with mental health support services [16].

**Server architecture.**

Frontend development

- React.js framework for responsive user interface
- Material-UI component library for consistent design
- WebSocket implementation for real-time chat functionality
- Client-side-only session management with no persistent storage

Backend infrastructure

- Node.js runtime environment with Express.js framework
- Stateless architecture with no database implementation
- Direct application programming interface (API) integration with OpenAI’s GPT-4
- Nginx reverse proxy for load balancing

OpenAI API integration

- Implementation of GPT-4 API with custom prompt engineering
- No retention of conversation history
- Each interaction is processed as a new, independent session
- Response token limiting for cost optimization
- Regular monitoring of API performance and reliability

### User-Centered Design and Emotion Monster Selection

The chatbot targets young adults, particularly university students aged 18-27 years. It uses a set of 25 emoticons based on emotion recognition research [15]. These emoticons allow users to express a wide range of emotions, similar to a broader palette of colors enhancing artistic appreciation. Recognizing this wider range of emotions can lead to greater self-awareness and sensitivity toward others, ultimately fostering empathy, connection, and understanding. When a user selects an emotion monster, a message appears on the chat screen stating their current mood. However, this message is not directly sent to the API. Instead, it is arbitrarily generated to include the selected emotion keyword and appear natural in the chat context. The actual message sent to the API is a combination of prompts, such as: “My current emotion is” + emotionText + “Acknowledge my feelings and greet me with a message of 50 characters or less.” The GPT engine receives this prompt-based message and generates a response reacting to the user’s selected emotion.

**Figure 2:**
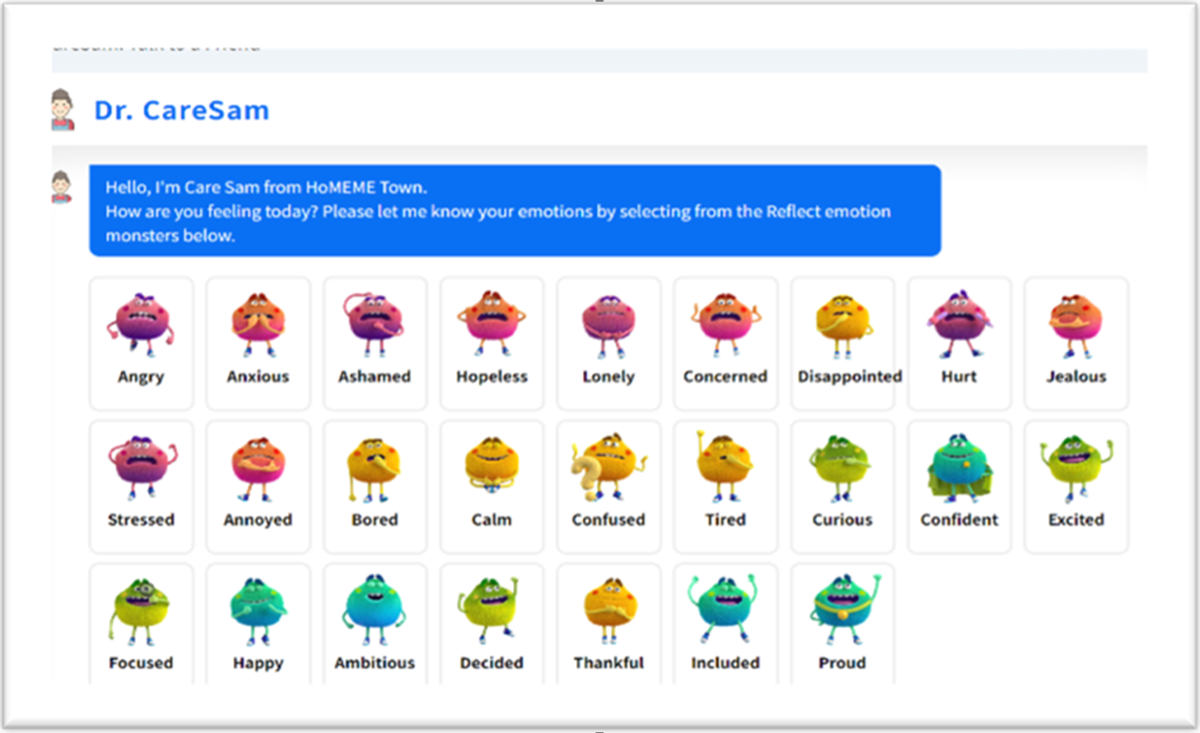
Emotional icons are used in the HoMemeTown chatbot.

Figure 2 displays the 25 emotional icons used in the HoMemeTown chatbot. Inspired by Microsoft’s emotion monsters, these icons represent a wide range of human emotions, allowing users to select one that reflects their current mood. This selection facilitates a more personalized and emotionally attuned response from the chatbot.

### Cross-Lingual Dialogue Development

The HoMemeTown chatbot was developed to support both English and Korean languages to cater to a wider audience. While our primary participants were Korean university students with intermediate to upper-intermediate English proficiency (based on self-reported language skills and academic records), they were able to effectively evaluate both language versions. The English version received higher ratings specifically in terms of written expression quality, though overall satisfaction levels were comparable between the 2 versions. This may be attributed to the more straightforward nature of emotional expression in English compared with the complex honorific system in Korean, as noted in our language-specific challenges.

In addition to our main study, we conducted informal preliminary feedback sessions with a native English speaker (not included in the formal participant count of 20). This native speaker reported high satisfaction with the natural flow and cultural appropriateness of the English version. While this supplementary feedback was exploratory in nature and limited in scope, it provided valuable initial insights into the cross-cultural applicability of our system.

The localization process involved adapting the chatbot’s dialogue to ensure natural conversation flow, accurate language translation, and cultural sensitivity [17]. The HoMemeTown chatbot was developed to support both English and Korean languages to cater to a wider audience. The localization process involved adapting the chatbot’s dialogue to ensure natural conversation flow, accurate language translation, and cultural sensitivity [17].

**Figure 3:**
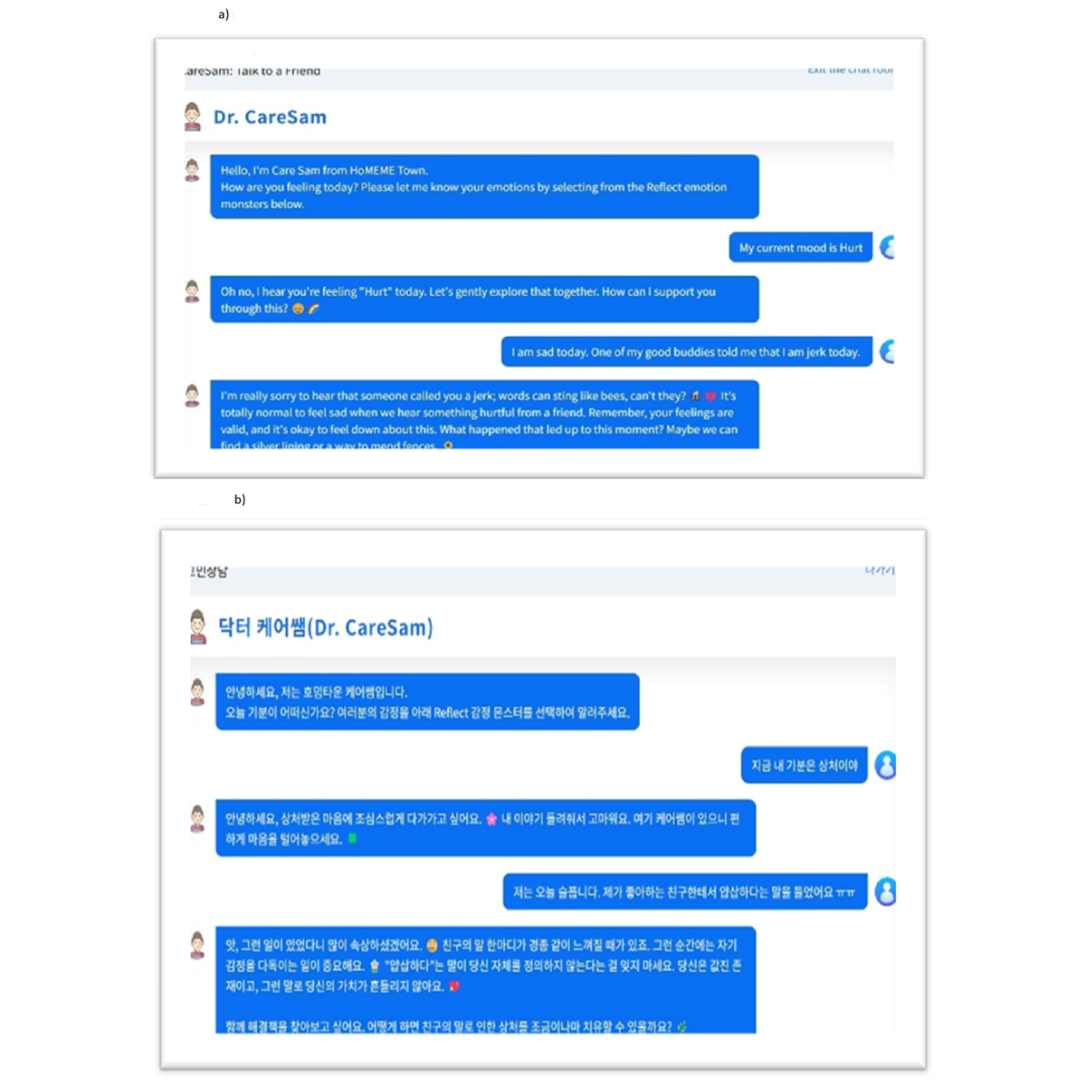
Dialogue flow localization. A: English version; B: Korean version.

Figure 3 illustrates the dialogue flow localization process for both English and Korean versions of the HoMemeTown chatbot. This visual representation demonstrates how the chatbot’s responses are adapted to maintain natural conversation flow and cultural appropriateness in each language.

The localization process is dialogue localization, language-specific challenges, and emoji usage.

#### Dialogue Localization

The chatbot’s dialogue was adapted for both English and Korean languages, taking into account language structure, emoji usage, and cultural expressions. The localization process aimed to maintain the chatbot’s empathetic and supportive tone while ensuring coherence and readability in each language. During the prompt tuning process, efforts were made to make the chatbot’s responses more flexible and engaging, particularly in the Korean version. For example, when a user expresses feeling down and asks for something fun to do, the Korean chatbot is tuned to provide humorous and entertaining responses, similar to the English version, instead of giving a rigid, therapist-like response.

#### Language-Specific Challenges

The Korean localization posed unique challenges due to its agglutinative nature and honorific system. In the Korean version of the HoMemeTown chatbot, Microsoft’s emotion adjectives were translated into Korean abstract nouns, such as “상처” (hurt), “외로움'”(lonely), “소속감” (inclusive), “실망” (disappointed), and “지루함” (bored). To maintain a consistent rule, these abstract nouns were combined with the verb ending “iya” (이야). However, this approach led to some awkward expressions, such as “상처이야” (hurt) and “자랑이야” (proud), while others, like “실망이야” (disappointed), “외로움이야” (lonely), and “지루함이야” (bored), sounded more natural. This inconsistency in the naturalness of the expressions highlights the complexity of the Korean language and the challenges in developing a chatbot that can generate linguistically accurate and culturally appropriate responses. Although collaborating with native Korean speakers and linguists helped ensure grammatical accuracy and appropriate honorific usage, further improvements are necessary to fully address the unique challenges posed by the Korean language, such as refining the translations and developing more sophisticated language-specific tuning techniques [17].

#### Emoji Usage

Emojis were strategically incorporated into both English and Korean dialogues to convey emotions and soften the tone of the conversation. The Korean dialogue used emojis more frequently to align with cultural communication preferences.

### Gratitude Journal

The gratitude journal feature was included in the prototype version based on positive feedback from our previous study [3]. In the previous year’s experiment, many students mentioned it as one of the most satisfying and enjoyable aspects of the app. Unlike the previous year’s app, where explanations of the effects and simple procedures for writing a gratitude journal were often omitted in Woebot and Happify, the user interface of the HoMemeTown chatbot’s gratitude journal includes a description of its effects and the procedure for using it.

The effects and procedures were based on established research [18], and the benefits of gratitude journaling have been demonstrated in numerous studies [13,14]. Users accumulate tokens for completed gratitude journal entries, viewable in their interface. To facilitate admin tracking of multiple user accounts, an admin page is implemented, allowing administrators to view gratitude journal entries, token balances, and chat frequencies for all user accounts.

**Figure 4:**
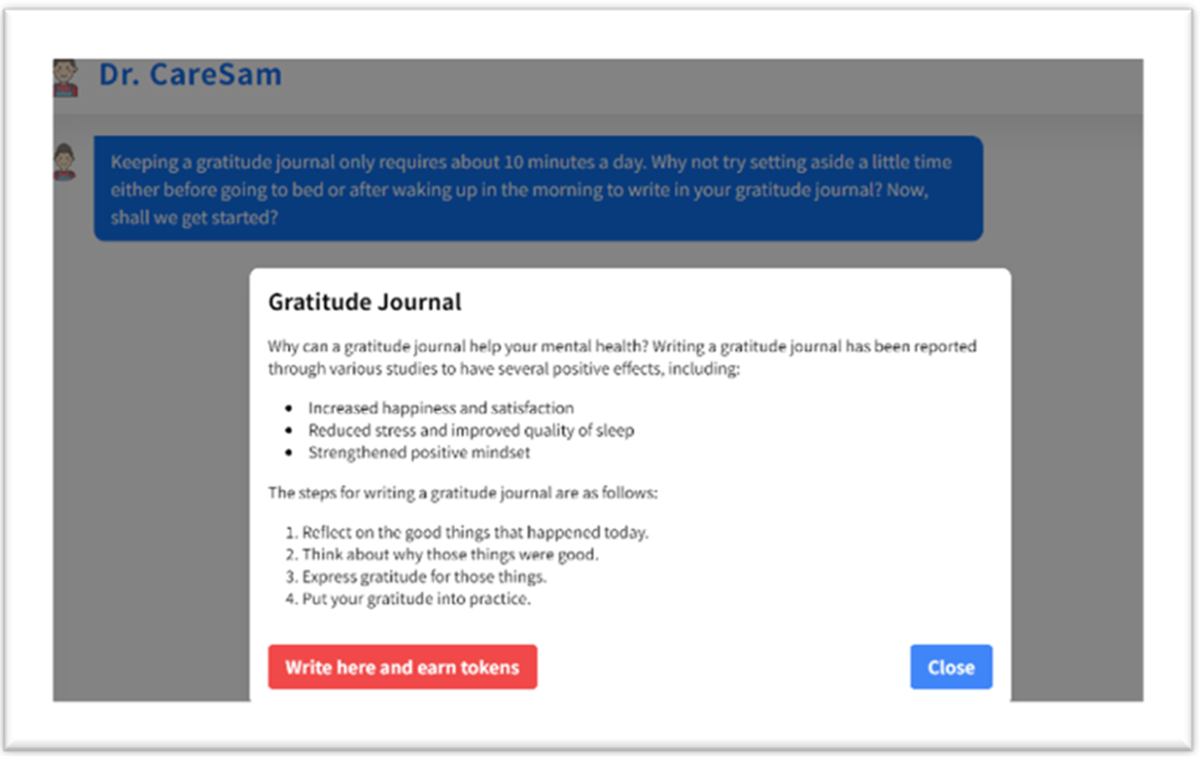
Gratitude journal interface in the HoMemeTown chatbot.

Figure 4 illustrates the gratitude journal interface in the HoMemeTown chatbot. The interface includes a description of the benefits of gratitude journaling and a step-by-step guide on how to write a gratitude journal entry. This feature encourages users to reflect on positive experiences, express gratitude, and cultivate a more optimistic mindset. Upon completing a journal entry, users are rewarded with tokens, which are displayed in the top-right corner of the chat interface.

### Risk Detection and Response System

The risk detection system in HoMemeTown was developed based on established clinical guidelines [19] and validated screening tools [20], implementing a sophisticated approach to identifying and responding to potential mental health concerns. The system continuously monitors user interactions for primary risk indicators, including expressions of suicidal ideation, severe depression symptoms, and anxiety crisis signals, while also tracking secondary indicators such as sleep disturbance patterns and social withdrawal signs.

When potential risks are detected, the system implements a graduated response protocol that has been carefully designed to provide appropriate levels of support while avoiding unnecessary escalation. For mild risk situations, the system offers empathetic acknowledgment and self-help resources, drawing from evidence-based interventions [21]. In cases of moderate risk, the response includes more direct expressions of concern and specific mental health resources, while severe risk triggers an immediate crisis response protocol with direct connections to professional support services.

To address the challenge of potential false positives in risk detection, we implemented a sophisticated validation system that examines multiple contextual factors before triggering interventions. This system uses NLP techniques to analyze the broader context of user communications, helping to distinguish between casual expressions and genuine indicators of distress. Regular professional review of high-risk cases ensures the ongoing refinement of detection algorithms and response protocols, maintaining a balance between sensitivity and specificity in risk assessment.

The risk detection and response system undergoes continuous evaluation and improvement based on user feedback and system performance metrics. Professional mental health experts regularly review the system’s performance, leading to protocol updates that reflect emerging best practices in digital mental health support. This iterative improvement process has been shown to enhance the accuracy and effectiveness of automated mental health support systems [22].

In addition, to mitigate variability and potential errors in LLM responses, we introduced a validation process including semantic consistency checks, medical reference verification, and automatic escalation to human review when necessary, ensuring responses remain clinically appropriate and user safety is maintained.

Our implementation of these technical and clinical safeguards reflects a balanced approach to leveraging AI capabilities while maintaining high standards of user safety and support quality. The system’s architecture and protocols were designed to be scalable and adaptable, allowing for continuous improvement based on ongoing research in digital mental health interventions. The HoMemeTown chatbot incorporates a risk detection function to identify potential mental health concerns through user interactions. This feature is based on the *DSM-5* (*Diagnostic and Statistical Manual of Mental Disorders* [Fifth Edition]) criteria [19] and a Korean corpus of psychopathological symptoms [20], allowing for culturally sensitive risk assessment.

The system monitors key symptoms such as depressed mood, changes in appetite or weight, sleep disturbances, fatigue, feelings of worthlessness, cognitive difficulties, and thoughts of death or suicide. When 3 or more symptoms are detected, the chatbot activates a response protocol to encourage professional help-seeking and provide relevant resources. As shown in Table 1, the response strategies are carefully adapted for cultural appropriateness in both English and Korean versions, with particular attention to different cultural norms in discussing mental health concerns.

In Table 2, it shows the differences that reflect the cultural variations in addressing mental health issues, highlighting the importance of culturally sensitive AI development in mental health applications. The use of emojis, while more prevalent in the Korean version, serves to soften the tone and enhance emotional expression in both languages, aligning with digital communication norms among young adults.

**Table 1:** Comparison of English and Korean chatbot versions.

Characteristic	English version	Korean version
Conversation style	Indirect, supportive	Direct, information-focused
Emotional approach	In-depth emotion exploration	Acknowledge emotions, quick transition
Introduction of professional help	Gradual	Immediate
Risk assessment method	Subtle progression	Explicitly stated
Cultural nuance	Emphasis on individual feelings	Focus on practical solutions

**Table 2:** Example risk detection responses with cultural adaptations.

Risk level	English response	Korean response
Mild	“I notice you're having a tough day 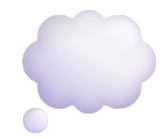 Would you like to talk about what's bothering you? 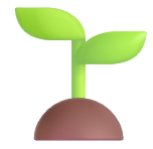 ”	“요즘 마음이 무거워 보이네요 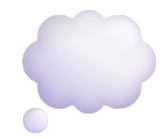 함께 이야기 나누면 도움이 될 수 있어요 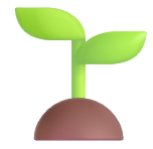 ”
Moderate	“It sounds like you're going through a difficult time 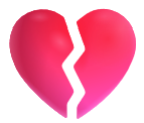 Have you considered talking to someone who can help? I can suggest some resources if you'd like 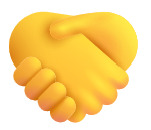 ”	“힘들어하시는 모습이 느껴져요 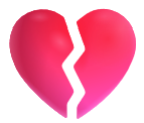 전문가와 상담해보는 건 어떠실까요? 제가 도움이 될 만한 정보를 알려드릴 수 있어요 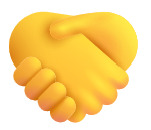 ”
Severe	“I'm very concerned about what you're sharing 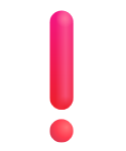 There are people available 24/7 who want to support you. Would you like the contact information for immediate help? 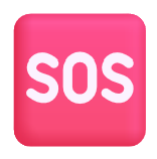 ”	“많이 걱정되는 이야기네요 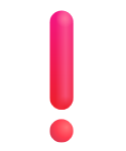 24시간 언제든 도움을 받으실 수 있는 곳이 있습니다. 지금 바로 연락하실 수 있는 곳을 알려드릴까요? 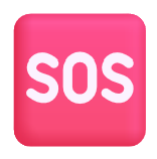 ”

### Ethical Considerations

This study was designed as an initial usability test of the HoMemeTown chatbot prototype, focusing on user experience and potential effectiveness. While it does not constitute a full-scale clinical intervention, we adhered to strict ethical guidelines for research involving human participants.

The study was conducted as part of a larger research project titled “Development of a Youth Mental Health Platform Using Natural Language Processing.” While the overarching project received initial institutional review board approval from Sungkyunkwan University (2023-02-043-004; February 27, 2023, to June 26, 2025), we acknowledge that this specific chatbot experiment was added later due to rapid developments in AI technology. Despite this limitation, we maintained rigorous ethical standards throughout our research, including informed consent, data privacy measures, and risk mitigation strategies.

Before the usability test, all participants were provided with a comprehensive informed consent form. This form detailed the nature and purpose of the study, the procedures involved, potential risks and benefits, and the measures taken to ensure confidentiality and data protection. Participants were required to sign this form to confirm their understanding and voluntary agreement to participate.

Approximately 70% of the participants in this study had previously participated in our earlier experiment [3]. This continuity helped streamline the consent process as these participants were already familiar with the ethical standards and procedures in place for digital mental health research.

### Data Security and Privacy

To address privacy concerns and protect sensitive information, the public version of the HoMemeTown chatbot operates without user registration or login requirements, collecting no personal information beyond chat interactions. This approach enhances user privacy but limits personalization features. For the usability test in this study, we implemented stringent data security measures, which are (1) all collected data (chat logs, gratitude journal entries, token rewards) were anonymized, (2) data were stored on secure, encrypted servers with restricted access, and (3) no personally identifiable information was linked to chatbot interactions or survey responses.

We incorporated a risk detection function to identify potential mental health crises and provide appropriate resources when necessary. These measures align with best practices in digital health research, ensuring ethical compliance and participant protection while advancing AI-assisted mental health support. Future developments will explore balancing personalization benefits with privacy protection, possibly through advanced encryption methods or privacy-preserving technologies.

## Results

### Overview

We conducted a mixed methods study to evaluate the performance and user satisfaction of our HoMemeTown Dr CareSam chatbot. The study design integrated quantitative and qualitative approaches to provide comprehensive insights: 8 quantitative questions (1 overall satisfaction item and 7 components of chatbot performance) and 4 qualitative questions (2 positive aspects and 2 areas for improvement). This mixed methods approach allowed for triangulation of data through cross-verification between quantitative metrics and qualitative user feedback, enhancing the validity and depth of our findings. The results provide multifaceted insights into the chatbot’s strengths, areas for improvement, and comparative performance with other LLM and digital therapy chatbots.

### Participants

The study included 20 participants aged 18 to 27 (mean 23.3, SD 1.96) years with 60% (12/20) female and 40% (8/20) male. Participants were recruited through university email lists and social media advertisements. All participants provided informed consent.

### Quantitative Findings

The usability and satisfaction evaluation of the Dr CareSam counseling chatbot was conducted using a comprehensive survey consisting of 1 overall satisfaction question and 7 quantitative items assessing key components of effective psychological counseling, which consists of empathy, accuracy and usefulness, complex thinking and emotions, active listening and appropriate questions, positivity and support, professionalism, and personalization. Users generally expressed positive views, with positivity and support receiving the highest score on a 10-point scale (mean 9.0, SD 1.2), followed by empathy (mean 8.7, SD 1.6) and active listening (mean 8.0, SD 1.8). These findings align with previous research on the importance of empathy and support in mental health chatbot interactions. However, areas for improvement were noted in professionalism (mean 7.0, SD 2.0), complexity of content (mean 7.4, SD 2.0), and personalization (mean 7.4, SD 2.4), indicating potential avenues for future development to enhance user engagement and satisfaction.

**Figure 5:**
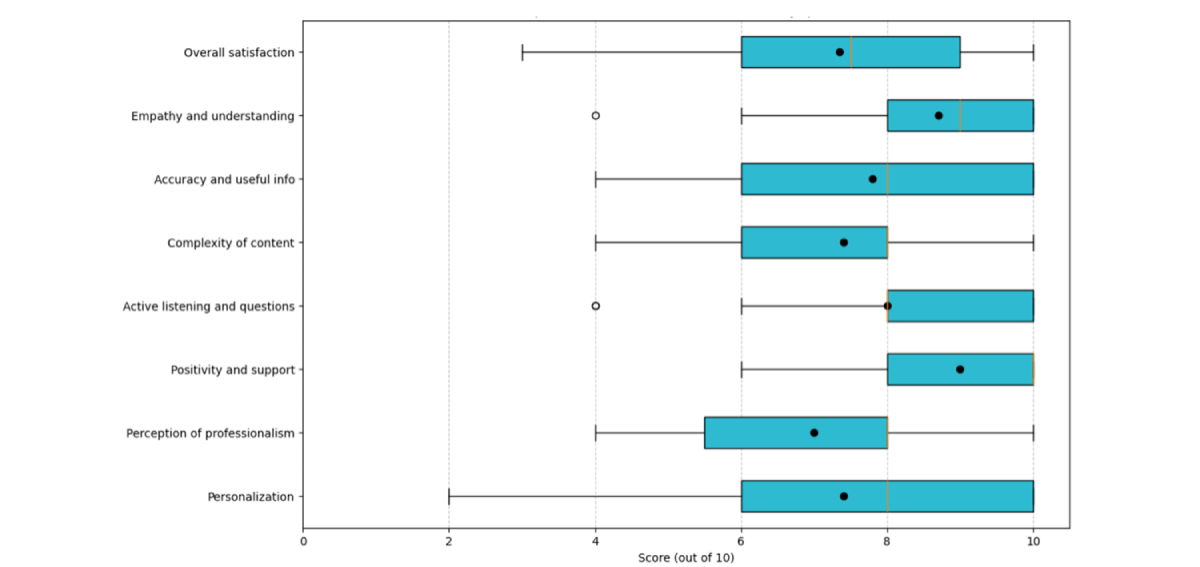
Box plot of scores for Dr CareSam usability questions.

Figure 5 shows the distribution of scores for various usability questions. The boxes represent the IQR, and the whiskers extend to the minimum and maximum values within 1.5 times the IQR. Black dots represent the mean values for each category.

To provide a more comprehensive understanding of the evaluation factors and their theoretical foundations, we present the following in Table 3.

This comprehensive evaluation framework enables a nuanced assessment of the chatbot’s performance across key dimensions of effective counseling. The results provide valuable insights into the strengths of the Dr CareSam chatbot, particularly in areas of empathy and support. In addition, the findings highlight opportunities for improvement in professionalism, personalization, and complexity of responses, suggesting potential avenues for future development to enhance user engagement and satisfaction.

**Table 3:** Evaluation Factors for Dr CareSam chatbot.

Evaluation factor	Question	Description	Previous studies
Empathy	Did CareSam’s responses express empathy and understanding of the user's question?	An empathetic understanding of the client's emotions and experiences is essential in effective counseling conversations.	Rogers [23], Elliott et al [24]
Accuracy and usefulness	Did CareSam’s responses provide accurate and useful information regarding the user’s question?	Providing accurate and actionable information aids in problem-solving and decision-making for the client.	Hepworth [25], Egan [26]
Complex thinking and emotions	Did CareSam’s responses include complex thinking and emotions rather than simple knowledge?	Skilled counselors should address cognitive and emotional experiences interactively, facilitating insights into the client's internal experiences.	Greenberg [27], Gendlin [28]
Active listening and appropriate questions	Did CareSam’s responses include not only active listening but also appropriate questions?	Active listening and appropriate questioning techniques promote self-exploration and deeper understanding.	Weger et al [29], Hill [30]
Positivity and support	Did CareSam’s responses include positivity and support?	Support and encouragement enhance the client's self-esteem and motivation for change.	Mearns and Thorne [31], Norcross and Lambert [32]
Professionalism	Did CareSam’s responses demonstrate professionalism?	Professionalism increases client trust and adherence to treatment, encompassing theoretical knowledge, clinical experience, and ethical awareness.	Sue and Sue [33]; Ratts et al [34]
Personalization	Did CareSam’s responses appear to be customized?	Effective counseling should be tailored to the individual characteristics and needs of the client.	Norcross and Wampold, [35], Beutler and Harwood [36]

### Qualitative Feedback

User feedback presented in Table 4 was collected through structured interviews and open-ended survey responses, focusing on key themes such as response speed, empathy, and personalization. Responses were categorized based on frequency of mention, providing a clear overview of commonly reported strengths and areas for improvement. User feedback confirms the chatbot’s strengths in providing detailed, empathetic responses, a user-friendly interface, and a supportive demeanor. However, areas for improvement include slow response times, Korean text flow issues, and repetitive interactions. Interestingly, both “quick feedback” and “slow response time” were frequently mentioned, suggesting that while feedback is relevant and timely within the context of a rule-based chatbot, it may not be the fastest possible response.

The qualitative themes align with quantitative satisfaction ratings, illustrating consistent patterns across user experiences. For instance, high ratings for “positivity and support” were reflected in user comments praising Dr CareSam’s empathetic responses. Conversely, lower ratings in “personalization” corresponded with feedback indicating a desire for more tailored interactions.

**Table 4:** Users’ experiences of good and bad points for HoMemeTown chatbots.

Mention level and category		Positive points	Negative points
**Most mentioned**			
	Response speed	Quick responses and real-time feedback.	Slow response time, lack of prompt responses.
	Friendly and Positive Tone	Encouraging, supportive responses, provides courage and understanding.	Overemphasis on empathy, repetitive responses without practical advice.
**Frequently mentioned**			
	Empathy capability	Expresses understanding and empathy effectively.	Focused too heavily on empathy without specific guidance or solutions.
	Korean language processing	Supports English and Korean; human-like, natural conversation flow.	Awkward phrasing in Korean, lack of accuracy and appropriateness in Korean responses.
**Moderately mentioned**			
	Detailed expression	Uses varied emotions and appropriate emoticons.	Repetitive tone, lack of personalization.
	Chatbot functionality	Emotional expression, gratitude journaling guidance, diverse responses for different situations.	Limited content, need for features like a reward for engagement, and the option to revisit previous interactions.
**Less mentioned**			
	Design and interface	Clean, user-friendly interface with intuitive design elements.	Issues with long text bubbles, lack of auto line breaks, and need for design improvements.
	Content and information	Provides relevant information and problem-solving advice.	Excessive use of emoticons, insufficient professional advice, and need for mobile accessibility improvement.
**Least mentioned**			
	Personalization	Positive and personalized responses, convey a warm and positive energy.	Insufficient personalization, lack of integration with medical or counseling services, missing human touch.
	Miscellaneous	Easy usability, approachable demeanor.	Inconsistent engagement incentives need for enhanced humanistic features.

### Comparative Analysis

**Figure 6:**
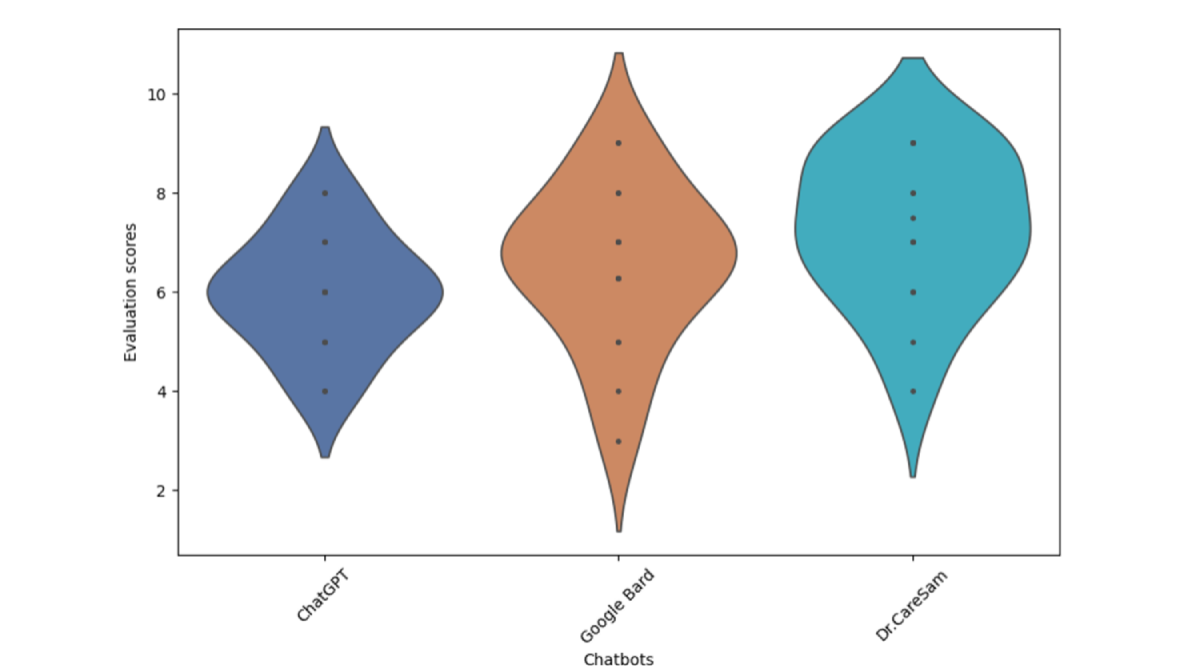
Comparison of large language models chatbots. LLM: Large language models.

Performance variations among LLM chatbots, including Google Bard and the freely accessible version of ChatGPT, as illustrated in Figure 6, were statistically significant (*F*=3.27; *P*=.048 While the evaluation primarily focused on “Overall Satisfaction” [3], it is important to note that user experience differences may reflect limitations inherent to the free versions available for these comparisons, including ChatGPT 3.0 and Google Bard’s publicly accessible iteration.

**Figure 7:**
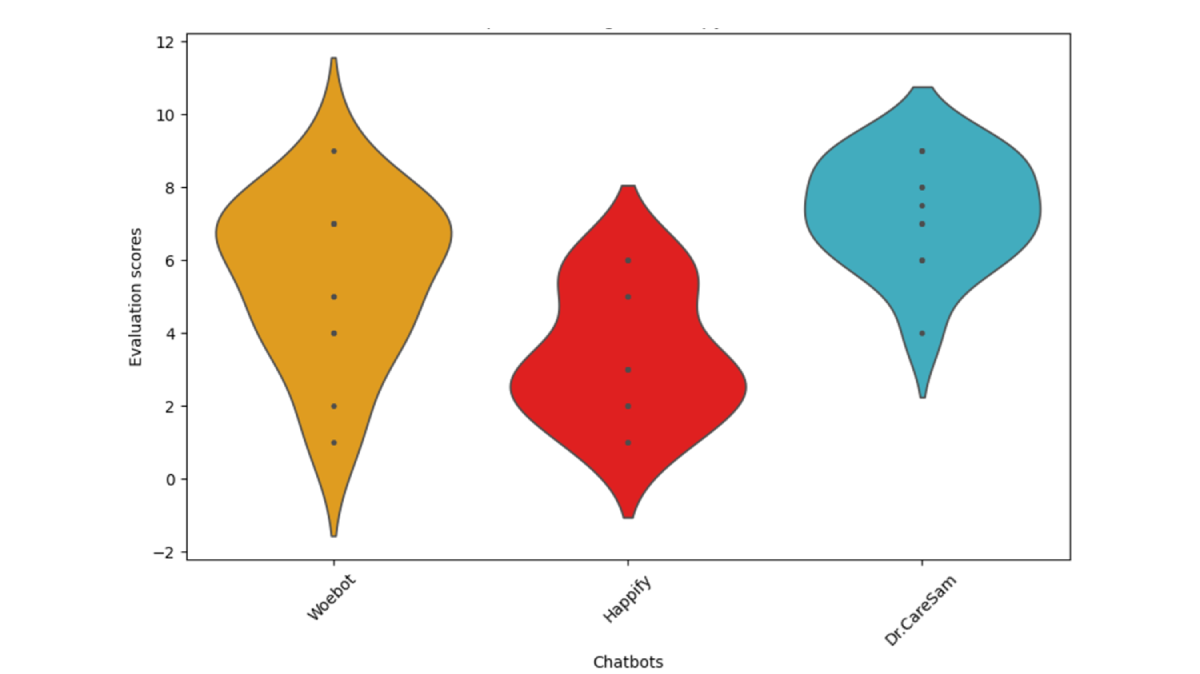
Comparison of digital therapy chatbots.

Satisfaction levels with HoMemeTown’s Dr CareSam, compared with Woebot [5] and Happify [37], showed more pronounced differences (*F*=12.94; *P*<.001), as shown in Figure 7, suggesting unique benefits in mental health support for college students.

In the comparison with Woebot and Happify, the previous evaluation [3] used 5 specific criteria, that are overall satisfaction, ease of use, novelty, effectiveness, and intention to maintain use. However, for this study, “Overall Satisfaction” was selected as the primary comparative metric to simplify and provide a focused assessment of user impressions. Statistical analyses were conducted using ANOVA to determine significant differences, with appropriate post hoc tests applied for multiple comparisons to identify specific group variations.

## Discussion

### Principal Findings

This study reveals several key insights about the HoMemeTown Dr CareSam chatbot, demonstrating its potential as an innovative tool in digital mental health support. The chatbot’s high performance across multiple dimensions of effective counseling, particularly in providing empathetic responses and a user-friendly interface, aligns with current research emphasizing the importance of these factors in digital mental health interventions [3,10,11]. The statistically significant performance differences observed between HoMemeTown Dr. CareSam and other chatbots, both LLM-based (*F*=3.27; *P*=.047) and traditional digital therapy tools like Woebot and Happify (*F*=12.94; *P*<.001), suggest that our approach offers unique benefits in mental health support for college students. This could be attributed to our comprehensive evaluation framework based on established counseling principles and the integration of a sophisticated risk detection function. The chatbot’s risk detection capability, grounded in *DSM-5* criteria and a Korean corpus of psychopathological symptoms, represents a significant advancement in AI-driven mental health support tools, enhancing its potential as a safe and responsible digital intervention.

However, the study also highlighted important challenges and areas for improvement. While the chatbot’s bilingual capability is a strength, issues with unnatural expressions and response speed in the Korean version underscore the complexities of cross-cultural adaptation in AI-driven mental health tools. Furthermore, our decision to prioritize user privacy over extensive personalization features reveals a critical challenge in developing ethical AI-driven mental health interventions. This trade-off between personalization and data protection warrants further exploration in the field. Specifically, areas for improvement were noted in professionalism (mean 7.0, SD 2.0), complexity of content (mean 7.4, SD 2.0), and personalization (mean 7.4, SD 2.4), indicating potential avenues for future development to enhance user engagement and satisfaction. Collectively, these findings suggest that HoMemeTown Dr CareSam represents a promising step forward in AI-assisted mental health support, while also illuminating critical areas for future research and development in this rapidly evolving field.

### Addressing LLM Variability and Technological Enhancements

To tackle the inherent challenges posed by LLM variability and potential hallucinations [38] in chatbot responses, we developed a comprehensive response validation pipeline. This pipeline includes semantic consistency checking, medical reference validation to prevent the dissemination of inaccurate information, and automatic escalation to human review when responses deviate from predetermined safety parameters. These safeguards are integral to ensuring the chatbot’s interactions remain clinically appropriate, fostering user trust and alignment with established mental health support practices.

While these measures provide a critical baseline for reliability, further advancements in the underlying LLM technologies are essential for achieving higher accuracy and contextual nuance in responses. For example, the integration of LangChain [39] technology allows for the systematic management and connection of multiple language models, offering improved contextual understanding and the ability to tailor responses to specific counseling scenarios. This modular approach enhances the flexibility and precision of chatbot interactions, particularly in complex or sensitive exchanges.

In addition, leveraging retrieval-augmented generation [40] techniques further bolsters response precision by drawing upon curated counseling databases and real-world cases. This not only strengthens the relevance of responses but also minimizes the risk of erroneous or hallucinated outputs. Such enhancements highlight the evolving interplay between foundational AI capabilities and domain-specific knowledge, positioning the chatbot as a more robust and dependable digital mental health intervention.

From an ethical standpoint, these advancements underscore the importance of balancing technological innovation with user safety and data integrity. Ensuring consistent oversight, ongoing evaluation, and refinement based on user feedback is vital to maintaining a high standard of care in digital interventions. As LLM technologies continue to evolve, our approach serves as a model for integrating emerging tools into practical applications, demonstrating how AI can be effectively harnessed to provide compassionate and reliable mental health support while continually adapting to user needs and technological developments.

### Comparison With Previous Work

Unlike rule-based chatbots, HoMemeTown Dr CareSam, leveraging LLM technology, was able to provide more flexible and personalized interactions. Our findings align with previous research on Woebot’s effectiveness in supporting young adults with depression and anxiety symptoms [5] while extending these benefits through our enhanced risk detection capabilities. Similarly, while Happify has shown promise in addressing loneliness during COVID-19 [37], our system demonstrates additional advantages in providing culturally adapted support for Korean users. This addresses several limitations of existing chatbots highlighted in previous studies, such as rigid response patterns and limited contextual understanding [3,5]. Specifically, our chatbot improved upon these limitations by offering more nuanced and context-appropriate responses, as evidenced by higher user satisfaction scores in empathy and active listening. The chatbot’s risk detection function, grounded in *DSM-5* criteria and a Korean corpus of psychopathological symptoms, represents an advancement in AI-driven mental health support tools, offering a level of clinical relevance not typically seen in general-purpose chatbots.

### Strengths and Limitations

A key strength of this study is the development of a personalized and empathetic mental health support tool using state-of-the-art LLM technology, with a sophisticated risk detection function. The bilingual support in English and Korean is another significant strength, addressing linguistic diversity and potential cross-cultural applications [17].

This study has several important limitations. The small sample size (n=20) limits the generalizability of the results but was chosen to ensure continuity and comparability with previous usability studies for Dr CareSam [3]. While this limited sample size may restrict broader applicability, it allowed for detailed and focused insights, particularly beneficial for pilot studies. Future research will aim to expand the sample size to validate findings and provide a more comprehensive evaluation of the chatbot’s effectiveness.

In addition, feedback from a small group of native English speakers, not formally included in the 20-participant sample, revealed potential areas for improving cross-lingual functionality. Though this feedback offered valuable preliminary insights, future studies will involve broader validation with a larger group of native speakers to ensure accurate and culturally appropriate responses.

Another limitation is the potential inconsistency in comparing Dr CareSam, built on the ChatGPT 4.0 API, with user experiences based on the freely available ChatGPT 3.0. Differences in capabilities between these versions may have influenced user perceptions and performance metrics. Future studies should strive to standardize versions for more direct and valid comparisons.

There are technical limitations associated with relying on the GPT API [7]. While this approach allows for advanced NLP capabilities, it also means that the chatbot’s performance is dependent on the underlying model, which may have inherent biases or limitations. Furthermore, the reliance on an external API raises considerations about data privacy and the long-term sustainability of the system.

These limitations highlight the need for large-scale, long-term studies to fully evaluate the chatbot’s effectiveness and generalizability. Future research should also explore the development of more specialized models that can be run locally, potentially addressing some of the limitations associated with relying on external APIs.

### Clinical Implications

The HoMemeTown Dr CareSam chatbot shows potential as an accessible mental health support tool for young adults, with its risk detection function providing an additional layer of safety. However, it’s crucial to clarify that this chatbot cannot replace professional mental health treatment, especially in cases where significant risk is detected. The chatbot’s role should be seen as complementary to traditional mental health services, potentially serving as an initial point of contact or a supplementary support tool. It may be particularly useful for providing immediate support during nonclinical hours, for mild to moderate concerns, or for individuals who may be hesitant to seek traditional face-to-face therapy. However, clear guidelines must be established for when and how to transition users from the chatbot to professional human intervention.

### Privacy and Personalization Considerations

A key challenge in this study was balancing personalization with user privacy. In our previous study, participants emphasized the importance of personalization features [3]. However, in developing the pilot version of HoMemeTown, we faced a significant dilemma between implementing these desired personalization features and ensuring robust privacy protection. Ultimately, we made the decision to prioritize privacy by eliminating user registration and personal data collection in the current public version. This choice was driven by the sensitive nature of mental health data and the potential risks associated with data breaches or misuse.

As a result, the chatbot relies solely on the capabilities of the LLM to provide a sense of personalization within individual conversations, without retaining user-specific information across sessions. This approach, while enhancing data security, limits our ability to offer some of the personalized features that participants in our previous study had requested. The chatbot attempts to mimic personalization through its conversational abilities, but it cannot retain or learn from past interactions with specific users. This trade-off highlights a crucial challenge in digital mental health interventions, how to balance user expectations for personalized experiences with the ethical imperative of protecting sensitive personal information. It also underscores the need for transparent communication with users about the capabilities and limitations of AI-driven mental health tools.

Future research should explore advanced technologies like federated learning or differential privacy, which could potentially allow for more personalized features without compromising user privacy. In addition, developing clear guidelines for handling mental health data in AI-powered interventions will be essential. Our experience underscores the need for innovative solutions that balance the benefits of personalization with robust data protection in mental health contexts. As the field evolves, finding this balance will be key to developing effective, trustworthy, and ethically sound AI-powered mental health interventions [8,41].

### Future Directions

Based on our findings, we propose the key areas for future research in Textbox 2.

These focused directions align closely with our current work while suggesting meaningful advancements in the field of AI-assisted mental health support within the context of medical informatics.

**Key Points of evaluation criteria for large language model (LLM) chatbots.**

Long-term effectiveness

- Conduct large-scale, longitudinal studies to evaluate the long-term impact of our cross-lingual chatbot on mental health outcomes.

Cross-cultural adaptations

- Further refine the chatbot’s ability to provide culturally appropriate responses, particularly focusing on improving Korean language naturalness. Improving the naturalness of Korean language responses will involve refining language processing algorithms and collaborating with linguists and native speakers to address issues with awkward phrasing and cultural nuance. Incorporating user feedback to continuously adapt and optimize the chatbot’s language output is also essential.

Speech-based user interface

- Develop and evaluate a speech-based user interface to increase usability and accessibility, particularly for users who may find voice interactions more natural or easier than text-based communication. This would involve integrating robust voice recognition and response capabilities to align with user preferences and accessibility needs.

Privacy-preserving personalization

- Explore technologies like federated learning to enhance personalization while maintaining robust data protection.

Risk detection enhancement

- Improve the accuracy and effectiveness of the risk detection function, potentially integrating it with existing mental health screening tools.

Integration with health care systems

- Investigate secure ways to integrate chatbot data with electronic health records, while maintaining user privacy.

### Conclusions

This study represents a significant advancement from our previous work [3], addressing the limitations identified and exploring the potential of more sophisticated AI technologies in mental health support. By leveraging ChatGPT 4.0 and incorporating features like cross-lingual support and risk detection, we have developed a more comprehensive and adaptable tool for supporting young adults’ mental health needs. This pilot study demonstrates the potential of HoMemeTown Dr CareSam, an LLM-based cross-lingual chatbot with advanced risk detection capabilities, in providing mental health support for young adults. While the chatbot showed promising results in user satisfaction, empathetic responses, and risk assessment, challenges in professionalism, cross-lingual adaptations, and the need for technical refinements were also identified. Further long-term, large-scale studies are needed to fully evaluate its effectiveness and potential integration with existing health care systems, as we continue to refine this technology to support mental well-being in our increasingly digital world. From a medical informatics perspective, this study contributes to our understanding of how advanced AI technologies can be applied in mental health care, potentially informing the development of more sophisticated, culturally sensitive digital health tools in the future.

## Abbreviations

AI: artificial intelligence
API: application programming interface
DSM-5: Diagnostic and Statistical Manual of Mental Disorders (Fifth Edition)
LLM: large language models
NLP: natural language processing
PHQ-9: Patient Health Questionnaire-9
